# Mindlin solution on ground deformation caused by the trench excavation during installation of concrete diaphragm wall panels

**DOI:** 10.1038/s41598-021-98403-z

**Published:** 2021-09-28

**Authors:** Guichun Zhu

**Affiliations:** 1 School of Rail Transportation, Soochow University, No. 8 Jixue Rd., Suzhou, 215131 China; 2 School of Civil Engineering, Yangzhou Polytechnic Institute, Yangzhou, 225000 China

**Keywords:** Civil engineering, Environmental impact

## Abstract

Concrete diaphragm walls (CDWs) are widely used as support of deep excavation in soft ground in urban areas. Ground deformation occurs during the excavation of trenches for the installation of the CDW panels due to ground stress release. This paper investigates the ground deformation during slurry trenching using Mindlin solution. The pressure of slurry used to protect the trench stability during excavation is simulated as a triangularly distributed load on the trench walls, the soil stress state is solved using Mindlin solution, and the horizontal and vertical ground displacement are obtained through integral transformation of ground strain. The rationality of the solution is verified through comparison between the analytical solution and field measurement. Sensitivity analyses are performed on soil elastic modulus, Poisson's ratio, trench excavation depth and panel length. Simplified formulation is proposed for the prediction of horizontal ground deformation. The impact of excavation stages and adjacent panels on the ground deformation is explored. The study finds that the Mindlin solution is able to provide an approximate solution on the ground deformation during slurry trenching, and the simplified formulation can be used to provide a fast estimate of the horizontal ground movement in engineering practice.

## Introduction

Concrete diaphragm walls (CDWs) are widely used as the support of deep excavation in soft ground in urban areas due to their high bending stiffness and excellent water tightness. As per the statistics from Liu et al.^1^, among the 760 subway stations constructed in the Yangtze River delta areas in southeast China, roughly 650 of them were supported by CDWs during the open-cut or cut-and-cover construction. The CDWs are constructed panel-by-panel with the typical panel length ranging from 4 to 6 m. The panels are installed by excavating a trench, placing a steel cage inside the trench, and tremie concreting the trench. During the trench excavation, bentonite slurry is filled inside to protect the wall stability. The typical unit weight of the slurry ranges from 1.05 to 1.25 g/cm^3^. The lateral slurry pressure is generally smaller than the original at-rest earth pressure, resulting in ground stress release and deformation during the slurry trenching process.

Field observations show that the slurry trenching induced ground settlement could account for 40 to 50% of the total ground settlement during the process of deep excavation supported by CDWs^2–4^. Clough and O’ Rourke^5^ early summarized the ground surface settlement data from five case projects of CDW installation and found that during slurry trenching, the average and maximum ground settlement adjacent to the wall reach about 0.08% and 0.15% of wall depth, respectively. Recently Mohamed^6^ summarized more case project data and plotted the ground settlement profile during slurry trenching. His profile is consistent with that of Clough and O’ Rourke^5^. Mohamed^6^ further plotted the lateral ground movement profile along wall depth. The lower and higher bound of lateral movement at ground surface next to the trench wall reach about 1 and 2 cm, respectively. Although the ground movement is relatively small and is typically neglected during the design and construction, it creaks risk for damage to adjacent structures, especially when the CDW is deep and the construction workmanship is poor. This portion of ground movement should be evaluated and considered during design and construction of deep excavation since the CDWs are typically used in sensitive construction environment.

The slurry trenching induced ground deformation was estimated by empirical method^7,8^, model testing^9–11^, numerical simulation^12,13^, and theoretical analysis^14–16^. The empirical method can obtain the shape diagram of the equilibrium arch and the limit equilibrium arch produced by the soil in the process of excavation and unloading, and it is proved that this arch effect becomes more and more obvious with the increase of deformation. It summarizes the field monitoring data and obtains the range of ground deformation using the statistical method to provide guidance to engineering practice. This method is simple, intuitive and widely used. This method, however, is limited by the high quality of field instrumentation data and can only be used to projects with similar ground conditions. The model testing can analyze the relationship between mud performance, liquid level, tank wall displacement, groundwater level and stability, establish the limit equilibrium mechanical analysis model and deduce the formula of safety factor. It is able to explore the mechanism of ground deformation but it is costly and difficult to conduct. The three-dimensional numerical simulation can study the stress–strain process of the trench wall soil during the whole excavation and concrete pouring process of the diaphragm wall. It is fast and cheap. However, the accuracy of the numerical simulation depends on the accuracy of the input geotechnical parameters which are difficult to characterize. The results of the numerical simulation need to be verified by other means. Theoretical analysis is able to obtain the general trend of the ground deformation and explore the sensitivity of the impacting factors. It provides an effective means to verify the numerical simulation results and reference to the model tests. Due to the complicated subsurface conditions and soil constitutive relationship, simplification needs to be made on the ground strata and soil properties to be able to derived a closed form solution.

Mindlin solution is utilized to solve the stress and strain in an elastic semi-infinite space under a concentrated force, especially pile foundations. The soil can be approximated as an elastic medium at a small strain state. As such, the Mindlin solution is able to be used to solve the stress and strain of soil under the action of external loads. With the rapid development of computer technology, Mindlin solution has been extensively used to solve practical problems in engineering. Based on Mindlin solution, Vaziri^17^ proposed a method for solving displacement under uniform load in semi-infinite space. Sun^18^ and Lei^19^ solved the surface settlement perpendicular to the center line of the trench section and verified it by three-dimensional finite element method, and analyzed the influence of the width, depth and excavation depth of the trench on the surface settlement. At present, the application of Mindlin solution is mostly in the calculation of vertical displacement. There are less reports on the application of Mindlin solution to the analysis of the horizontal deformation of the soil under specific conditions.

In this paper, the horizontal concentrated force in Mindlin solution is transformed into a triangular distributed load, which is applied to the side of the diaphragm wall to simulate the difference between the slurry pressure and the static lateral soil pressure. Then, the Mindlin solution is integrated to calculate the horizontal and vertical displacement of the surrounding soil caused by the construction of the diaphragm wall in the semi-infinite homogeneous stratum.

## Mindlin solution

**Figure 1:**
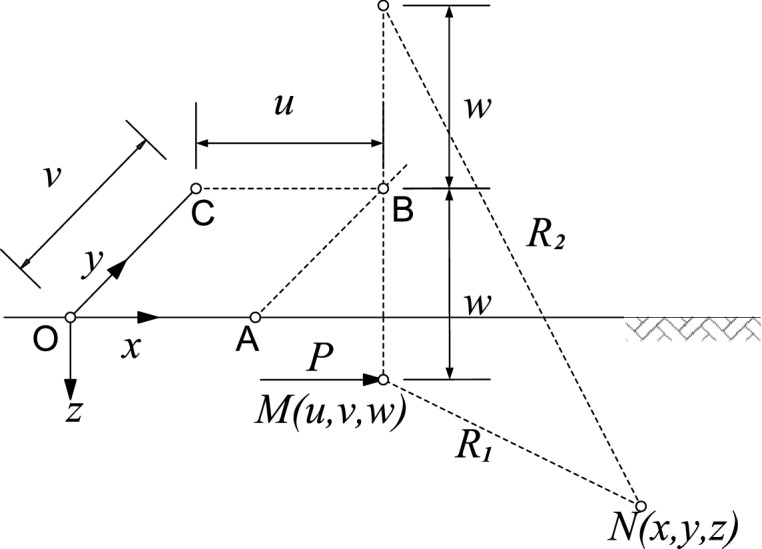
Coordinate system (OABC lies on the surface of the half-space).

Applying Mindlin solution^20^, within an elastic infinite half space, the horizontal displacement *δ*_*x*_ and vertical displacement *δ*_*z*_^18^ at any location *N*(*x*, *y*, *z*) under the action of a horizontal concentrated load *P* at any location *M*(*u*, *v*, *w*) can be expressed in Eqs. (1) and (2), The schematic view of location *N* and *M* is shown in Fig. 1.

1 $$ \delta_{{\text{x}}} = \frac{P}{\beta }\left\{ {\left( {3 - 4\nu } \right)\left( {\frac{1}{{R_{1} }} + \frac{{X^{2} }}{{R_{2}^{3} }}} \right) + \frac{1}{{R_{2} }} + \frac{{X^{2} }}{{R_{1}^{3} }} + \frac{2wz}{{R_{2}^{3} }}\left( {1 - \frac{{3X^{2} }}{{R_{2}^{2} }}} \right)} \right.\left. { + \frac{{4\left( {1 - \nu } \right)\left( {1 - 2\nu } \right)}}{{R_{2} + Z_{2} }}\left[ {1 - \frac{{X^{2} }}{{R_{2} (R_{2} + Z_{2} )}}} \right]} \right\} $$

2 $$ \delta_{z} = \frac{PX}{\beta }\left[ {\frac{{\left( {3 - 4\nu } \right)Z_{1} }}{{R_{2}^{3} }} + \frac{{Z_{1} }}{{R_{1}^{3} }} - \frac{{6wzZ_{2} }}{{R_{2}^{5} }} + \frac{{4\left( {1 - \nu } \right)\left( {1 - 2\nu } \right)}}{{R_{2} (R_{2} + Z_{2} )}}} \right] $$

where: *E* is Young’s modulus, $\nu$ is Poisson's ratio, $\beta = 8\pi E\frac{1 - \nu }{{1 + \nu }}$, $R_{i} = \sqrt {X^{2} + Y^{2} + Z_{i}^{2} } (i\, = \,1 \, or \, 2),X = x - u,Y = y - v,Z_{2} = z + w,Z_{1} = z - w,$

### Horizontal displacement

Assuming the slurry and groundwater at the same level at the ground surface, the slurry pressure follows a triangular distribution from the ground surface along depth. Within a certain range of depth below the ground surface, the slurry pressure follows a trapezoidal distribution on the trench wall to balance the lateral hydrostatic and soil pressure. The unit force pressure on the side of the diaphragm wall is shown in Eq. (3).

3 $$ P = \frac{1}{{(w_{2} - w_{1} )}}\left[ {(w_{2} p_{1} - w_{1} p_{2} ) + (p_{2} - p_{1} )w} \right]dvdw $$

**Figure 2:**
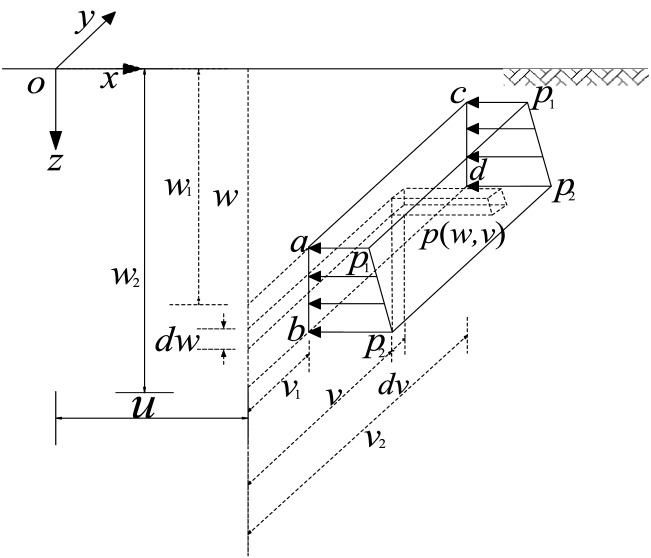
Horizontal pressures over a vertical rectangle.

The parameters in the Eq. (3) are presented in Fig. 2. *w*_*1*_, *w*_*2*_ are the upper and lower limits of the slurry depth range, *v* is the wide range, *p*_*i(i*=*1, 2*)_ is the difference between slurry pressure and lateral static earth pressure, as shown in Eq. (4).

4 $$ p_{i} = K_{0} (\gamma_{sat} - \gamma_{w} )z + (\gamma_{w} - \gamma_{s} )z,(i\, = \,1,2) $$

where, *γ *_*s*_ is unit weight of slurry, *γ*_*sat*_ is the saturated unit weight of soil, *γ *_*w*_ is the unit weightof water, and *K*_*0*_ is the coefficient of at-rest earth pressure.

As shown in Fig. 2, the Eqs. (1) and (3) are integrated on the bounded closed region *U*(*v*_*1*_ ≤ *v* ≤ *v*_*2*_, *w*_*1*_ ≤ *w* ≤ *w*_*2*_), the results are shown in Eq. (5).

5 $$ \delta_{x} = \frac{{\left( {p_{2} - p_{1} } \right)}}{{\beta \left( {w_{2} - w_{1} } \right)}}\left[ {\left\{ {G1_{x} (w,v)} \right\}} \right.\left. {_{{w = w_{1} }}^{{w = w_{2} }} } \right]_{{v = v_{1} }}^{{v = v_{2} }} + \frac{{\left( {p_{1} w_{2} - p_{2} w_{1} } \right)}}{{\beta \left( {w_{2} - w_{1} } \right)}}\left[ {\left\{ {G2_{x} (w,v)} \right\}} \right.\left. {_{{w = w_{1} }}^{{w = w_{2} }} } \right]_{{v = v_{1} }}^{{v = v_{2} }} $$

where:

6 $$ \begin{aligned} G1_{x} (w,v) & = \left( {3 - 4\nu } \right)\left\{ {\log \left( {R_{1} + Y} \right)\left( {\frac{1}{2}\left( {X^{2} + Z_{1}^{2} } \right) + zZ_{1} } \right)} \right. + Yz\log \left( {R_{1} + Z_{1} } \right) + \frac{{R_{1} Y}}{2}  \hfill \\ & \quad + \left. {X\left( {wT_{x2} + zT_{x1} + zT_{xy} } \right) - vz} \right\} + \frac{1}{2}\left( {Z_{1} Z_{2} - X^{2} } \right)\log \left( {R_{2} + Y} \right) - Yz\log \left( {R_{2} + Z_{2} } \right)  \hfill \\ & \quad + \frac{{R_{2} Y}}{2} - Xz\left( {T_{xy} - T_{x2} } \right) - XZ_{2} T_{x2} + Yz + \left\{ {zR_{2} \left( {\frac{{w^{2} X^{2} Y}}{{R_{2}^{2} Z_{2} \left( {X^{2} + Z_{2}^{2} } \right)}} - } \right.} \right.\left. {\frac{{Yz^{2} }}{{Z_{2} \left( {X^{2} + Y^{2} } \right)}}} \right)  \hfill \\ & \quad+ z(Y\log \left( {R_{2} + Z_{2} } \right) + \left( {Z_{1} + Z_{2} } \right)\log \left( {R_{2} + Y} \right) + \left. {XT_{xy} - v - 2XT_{x2} )} \right\}  \hfill \\ & \quad - \frac{{2\left( {1 - \nu } \right)\left( {1 - 2\nu } \right)}}{3}\left\{ {\left( {3\left( {Yz + YR_{2} } \right. + } \right.} \right.\left. {\left. {\frac{{\left( {2w - z} \right)Z_{2}^{2} \left( {T_{x2} - Z_{2} } \right)}}{X}} \right) - 3zZ_{2} \log \left[ {Y + R_{2} } \right] - 3Y} \right)  \hfill \\ & \quad \times z\log \left[ {Z_{2} + R_{2} } \right] + \left( {3wZ_{2} + X^{2} } \right)\log \left[ {Y + R_{2} } \right] + \frac{{Y\left( {2w - z} \right)\left( {w + z} \right)\left( {R_{2} - w - z} \right)}}{{\left( {X^{2} + Y^{2} } \right)}}  \hfill \\ & \quad - \frac{{\left( {2w - z} \right)\left( {w + z} \right)^{2} T_{xy} }}{X} - \left. {\frac{{\left( { - 2w^{3} - 3w^{2} z + 3X^{2} z + z^{3} } \right)T_{x2} }}{X} - \frac{{2\log \left[ {Y + R_{2} } \right]}}{{X^{2} }}} \right\} \, \hfill \\ \end{aligned} $$

7 $$ \begin{aligned} G2_{x} (w,v) & = \left( {1 - 2\nu } \right)\left( {\frac{{R_{2} XT_{2X} }}{{R_{2} }} + Y\log \left( {R_{1} + Z_{1} } \right) + Z_{1} } \right. \times \log \left( {R_{1} + Y} \right) - w + XT_{{xz_{1} }} + \left. {\frac{{X^{2} YT_{1x} }}{XY}} \right) \hfill \\ & \quad + Y\log \left( {R_{2} + Z_{2} } \right) + Z_{2} \log \left( {R_{2} + Y} \right) + XT_{xy} + XT_{1x} - XT_{2x} - v  \hfill \\ & \quad+ 2z \times \left( {\frac{{R_{2} Yz}}{{Z_{2} \left( {X^{2} + Y^{2} } \right)}} - \log \left( {R_{2} + Y} \right) + \frac{{wXT_{2x} }}{{Z_{2}^{2} }}} \right) + 2\left( {1 - \nu } \right)\left( {1 - 2\nu } \right)  \hfill \\ & \quad \times \left( {\frac{{\left( {Z_{2}^{2} - X^{2} } \right)\left( {T_{x2} - T_{xy} } \right)}}{X}} \right. - v + 2Z_{2} \log \left[ {Y + R_{2} } \right]Y\log \left[ {Z_{2} + R_{2} } \right] \hfill \\ & \quad \left. { - \frac{{Y\left( {R_{2} - Z_{2} } \right)Z_{2} }}{{X^{2} + Y^{2} }} + \frac{{Z_{2}^{2} T_{xy} }}{X} - \frac{{\left( {Z_{2}^{2} + X^{2} } \right)T_{x2} }}{X}} \right) \hfill \\ \end{aligned} $$

where: $T_{\alpha i} = \arctan \frac{{XYZ_{i} }}{{\alpha^{2} R_{i} }}$ (*α* = *X*, *Y* or *Z*_*i*_; *i* = 1 or 2) ; $T_{AB} = \arctan \frac{B}{A}$.

According to the concept of double definite integral,

8 $$ \left[ {\left\{ {Gi_{m} (w,v)} \right\}} \right.\left. {_{{w = w_{1} }}^{{w = w_{2} }} } \right]_{{v = v_{1} }}^{{v = v_{2} }} = Gi_{m} (w_{1} ,v_{1} ) + Gi_{m} (w_{2} ,v_{2} ) - Gi_{m} (w_{1} ,v_{2} ) - Gi_{m} (w_{2} ,v_{1} ),(i\, = \,1,2;m\, = \,x,y) $$

Eqs. (5) to (7) can be solved according to Eq. (8), and the horizontal displacement under the horizontal trapezoidal distributed load in semi-infinite space can be obtained.

### Vertical displacement

9 $$ \delta_{z} = \frac{{\left( {p_{2} - p_{1} } \right)X}}{{\beta \left( {w_{2} - w_{1} } \right)}}\left[ {\left\{ {G1_{z} (w,v)} \right\}} \right.\left. {_{{w = w_{1} }}^{{w = w_{2} }} } \right]_{{v = v_{1} }}^{{v = v_{2} }} + \frac{{X\left( {p_{1} w_{2} - p_{2} w_{1} } \right)}}{{\beta \left( {w_{2} - w_{1} } \right)}}\left[ {\left\{ {G2_{z} (w,v)} \right\}} \right.\left. {_{{w = w_{1} }}^{{w = w_{2} }} } \right]_{{v = v_{1} }}^{{v = v_{2} }} $$

where:

10 $$ \begin{aligned} G1_{z} (w,v) & = \left( {4\nu - 3} \right)\left( {\left( {3z\ln \left( {R_{2} + Y} \right) + Y\ln \left( {R_{2} + Z_{2} } \right) - v} \right) - } \right.\left. {\frac{{T_{x2} \left( {X^{2} - 2z^{2} } \right)}}{x} + XT_{xy} } \right)  \hfill \\ & \quad- { 2}z\left( { - \frac{{w^{2} Y}}{{R_{2} \left( {X^{2} + Z_{2}^{2} } \right)}} - 2\ln \left( {R_{2} + Y} \right)} \right.\left. { - \frac{{2zT_{x2} }}{X}} \right) + 2\left( {\nu - 1} \right)\left( {2\nu - 1} \right)\left( {2z\ln \left( {R_{2} + Y} \right)} \right.  \hfill \\ & \quad \left. { +  \frac{{\left( {Y\log \left( {R_{2} + Z_{2} } \right) - v} \right) + \left( {X^{2} - Z_{1} Z_{2} } \right)\left( {T_{xy} - T_{x2} } \right)}}{X}} \right)  \hfill \\ & \quad + \left( {z\ln \left( {R_{1} + Y} \right) + Y\ln \left( {R_{1} + Z_{1} } \right) + XT_{x1} - XT_{xy} + v} \right) \hfill \\ \end{aligned} $$

11 $$ \begin{aligned} G2_{z} (w,v) & = 2z\left( {\frac{wY}{{R_{2} \left( {X^{2} + Z_{2}^{2} } \right)}} - \frac{{T_{x2} }}{X}} \right) + 4\left( {\nu - 1} \right)\left( {2\nu - 1} \right)\left( {\frac{{Z_{2} \left( {T_{xy} - T_{x2} } \right)}}{X} - \ln \left( {R_{2} + Y} \right)} \right)  \hfill \\  & \quad + \left( {\left( {3 - 4\nu } \right)\left( {\ln \left( {R_{2} + Y} \right) + \frac{{2zT_{x2} }}{X}} \right) + \ln \left( {R_{1} + Y} \right)} \right) \hfill \\ \end{aligned} $$

The parameter definition in the equation is the same as that in Eq. (7). According to Eq. (8), the vertical displacement of Eq. (9) to (11) under horizontal trapezoid distributed load in semi-infinite space can be achieved.

### Validation

**Figure 3:**
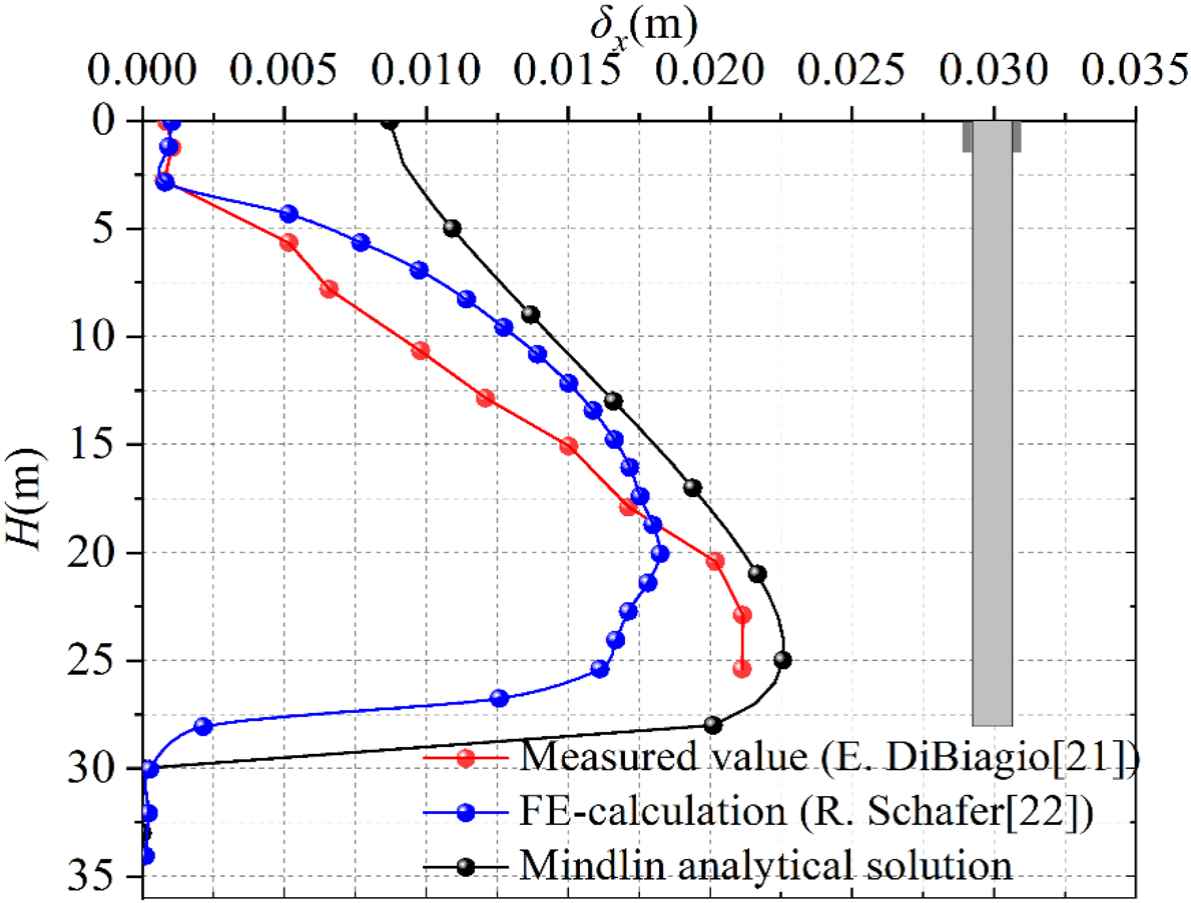
Horizontal displacement comparison results (along the vertical centerline).

**Figure 4:**
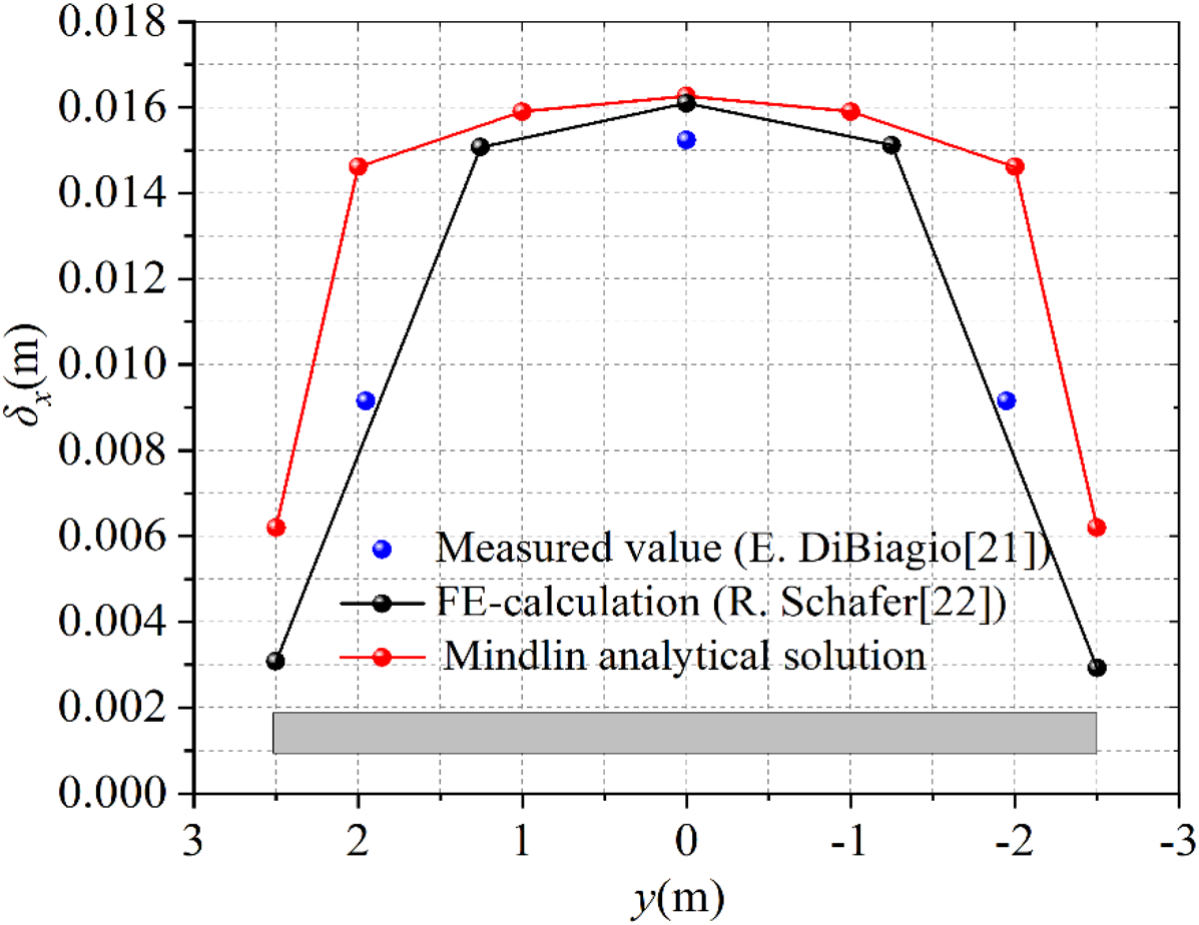
Horizontal displacement comparison results (at a depth of 15.5 m).

In order to verify the validity of the Mindlin solution, this paper takes the full scale field test of a slurry-trench excavation in Oslo, Norway as the background^21^, which especially investigated the deformations of the surrounding ground and the stability of the trench during the trench excavation and a subsequent resting period under slurry support, There were 14 gauges placed to determine soil movements, 10 along the vertical centerline of the trench and 5 in a horizontal plane at a depth of 15.5 m. R. Schafer generated a three-dimensional finite-element model for the simulation of the slurry-trench excavation in Oslo^22^. In this paper Mindlin solution is compared with the measurements of the slurry trench test excavation in Oslo^21^ and the calculated soil movements^22^. Horizontal displacement comparison along the vertical centerline of the trench is shown in Fig. 3, and horizontal movements at a depth of 15.5 m is shown in Fig. 4.

The test panel shows a depth of *D* = 28 m, a length of *L* = 5 m and a width of *W* = 1 m and was excavated using a slurry with an unit weight of 10.8 kN/m^3^. Parameter “H” is excavation depth of the trench. The test panel was in a clay-filled canyon which is typical of the Oslo region, most of soil layer parameters at the site are determined by different in situ and laboratory testings. *K*_*0*_, coefficient of at-rest earth pressure, was measured by the hydraulic fracturing method at different elevations. The weighted average values of the parameters of each soil layer are considered as *γ* = 18.8 kN/m^3^, *E* = 20 MPa, *v* = 0.35, *K*_*0*_ = 0.65, a more detailed description is given by R. Schafer^22^.

The Finite-element model generated by R. Schafer consisted of about 7500 trilinear brick elements incorporating a coupled calculation of the pore water flow and the effective stress field.

It can be seen from Fig. 3, Mindlin analytical solution is of vertical spoon shape. Because of the trench excavation, the horizontal deformation of the soil increasd with the depth of the trench. The measured maximum displacement occurs at 25.4 m, the displacement is 21.1 mm, and the maximum displacement of Mindlin solution occurs at 24 m, about 5/6 *D* (*D* is the depth of the groove), the maximum displacement is 22.57 mm, the position which the maximum vertical displacement occured is similar, and the maximum displacement error is 6.5%. It can get that the Mindlin analytical solution agrees well with the FE- calculation by R. Schafer^22^ and the change trend of the field measured value^21^.

Figure 4 illustrates the horizontal displacement comparison of the trench side at a depth of 15.5 m. It can be seen from Fig. 4, the horizontal displacement (at a depth of 15.5 m) presents a U-shaped curve with large displacement in the middle and small displacement on both sides along the long side of the trench. The maximum displacement occurs at *y* = 0. The measured maximum displacement is 15.23 mm, the maximum displacement of Mindlin solution is 16.26 mm, and the maximum displacement of finite element solution is 16.09 mm. The positions of these three maximum displacements are similar, and the maximum displacement error is 6.3%. It can be observed that the Mindlin analytical solution is in good agreement with the measured value and FE- calculation.

The above analysis shows that it is feasible to use Mindlin solution to solve the formation deformation caused by the trench formation of concrete diaphragm wall.

## Sensitivity factors of horizontal displacement

The horizontal displacement is related to the elastic modulus *E*, Poisson's ratio *v*, the excavation depth *D*, the excavation length *L*, the slurry density *γ*_*s*_, and the saturated gravity density *γ*_*sat*_. Now, it is set that *E* = 20 MPa, *v* = 0.49, *D* = 45 m, *L* = 6 m, *γ*_*sat*_ = 20kN / m^3^, *γ*_*w*_ = 10kN/m^3^, *γ*_*s*_ = 11 kN/m^3^. The single variable method is used to analyze the influence of each parameter on the horizontal displacement of concrete diaphragm wall.

### Elastic modulus and Poisson's ratio

**Figure 5:**
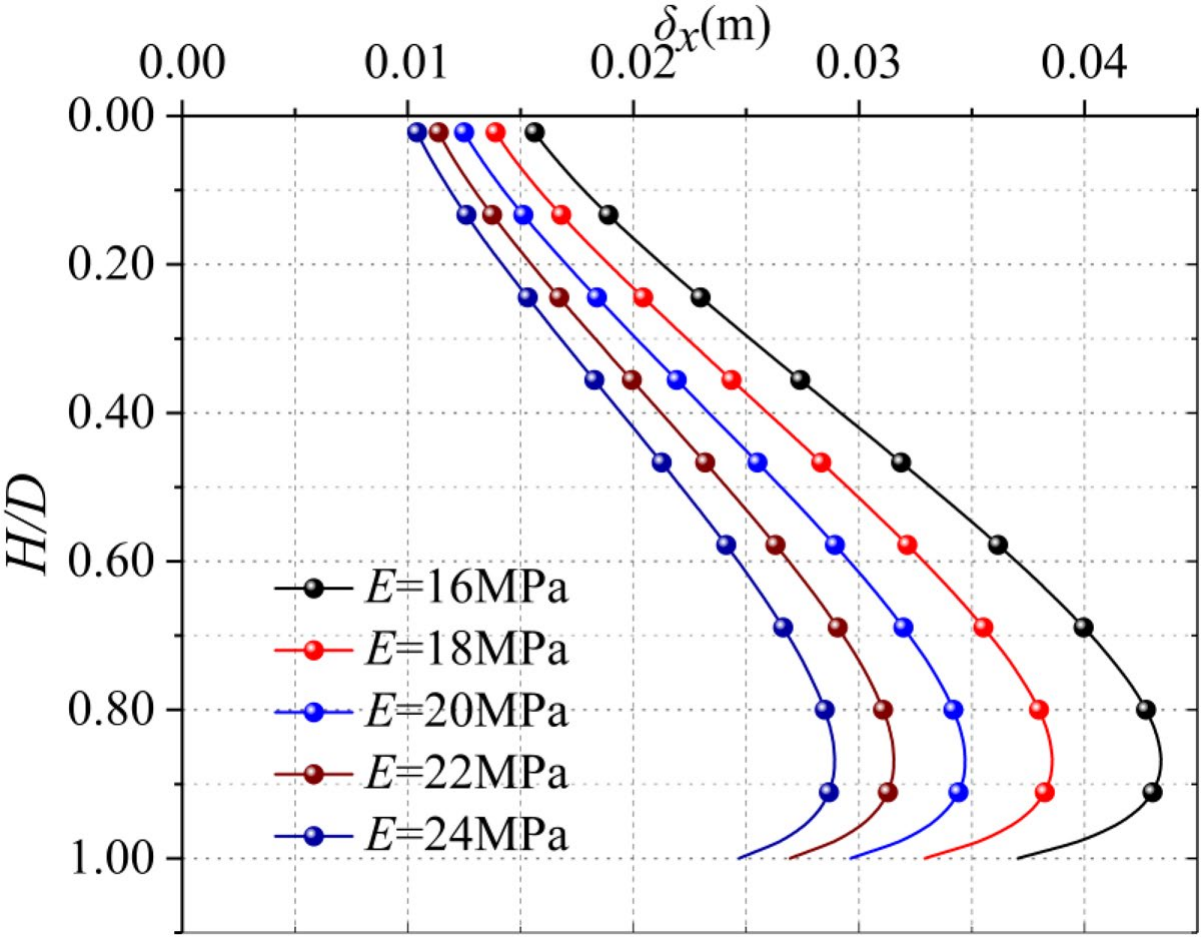
Horizontal displacement with different elastic modulus.

**Figure 6:**
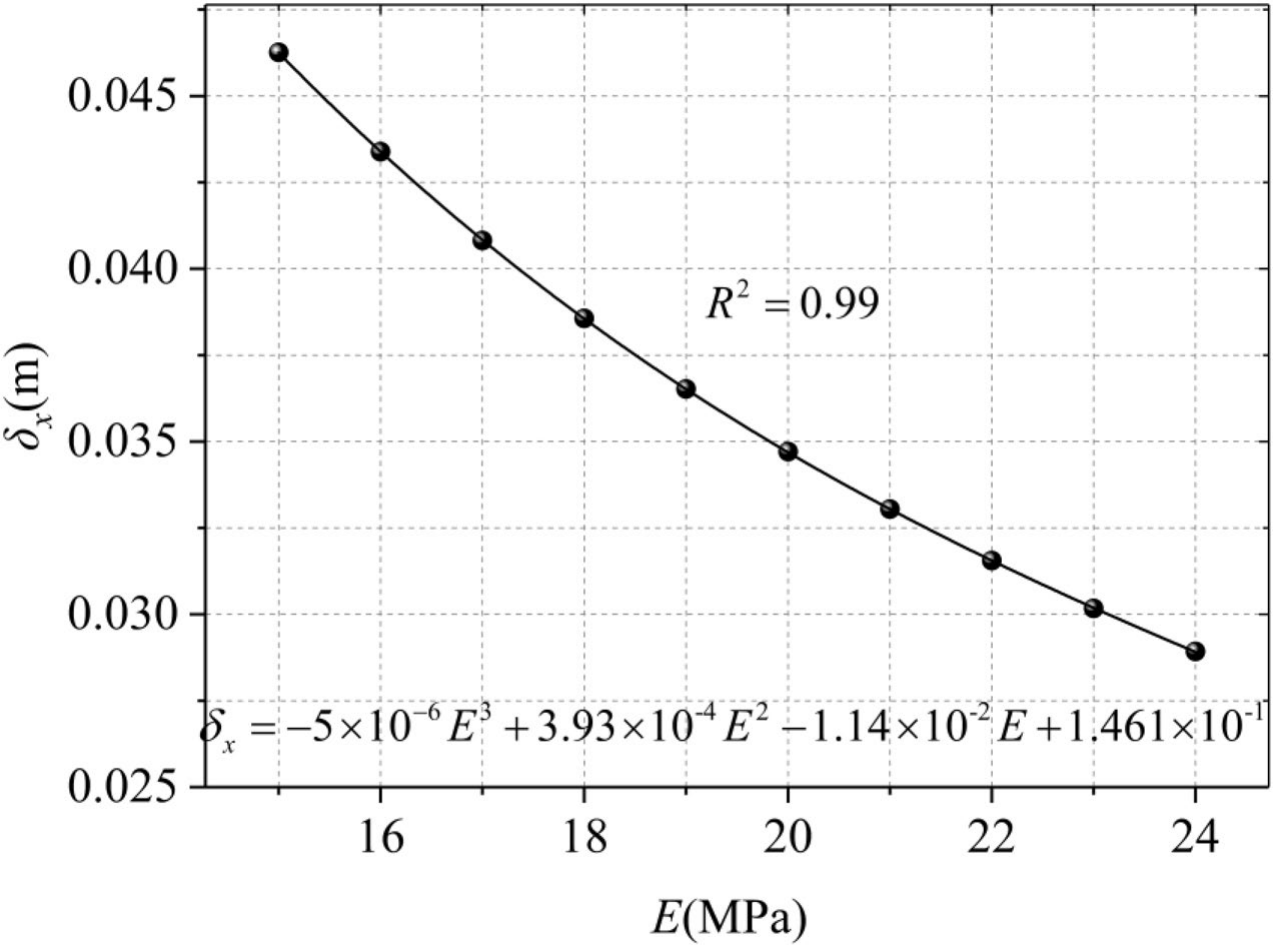
Maximum horizontal displacement with different elastic modulus.

Modulus of elasticity is a key parameter of soil, which directly reflects the relationship between stress and strain. It can be seen from Fig. 5, the maximum horizontal displacement occurs at about 5/6 *D*. The maximum horizontal displacement decreases with the increase of Elastic modulus, and the reduction rates are 12.50%, 11.11%, 10.01% and 9.09% respectively. It can be observed that under the same working condition, the larger the elastic modulus is, the smaller the influence of disturbance on the soil is, and the smaller the horizontal displacement of the soil around the trench wall is. The relationship between the maximum horizontal displacement and the modulus of elasticity is nonlinear, as shown in Fig. 6, and the relationship can be roughly simulated as shown in Eq. (12):

12 $$ \delta_{x} \, = - 5 \times 10^{ - 6} E^{3} \, + { 3}{\text{.93}} \times 10^{ - 4} E^{2} \, - \, 1.14 \times 10^{ - 2} E \, + \, 1.461 \times 10^{ - 1} $$

It can be observed in Fig. 6 that when *E* is small, the maximum horizontal displacement of soil increases rapidly with the increase of *E*, while when *E* increases to a certain extent, the growth rate slows down and the parameter sensitivity weakens.

**Figure 7:**
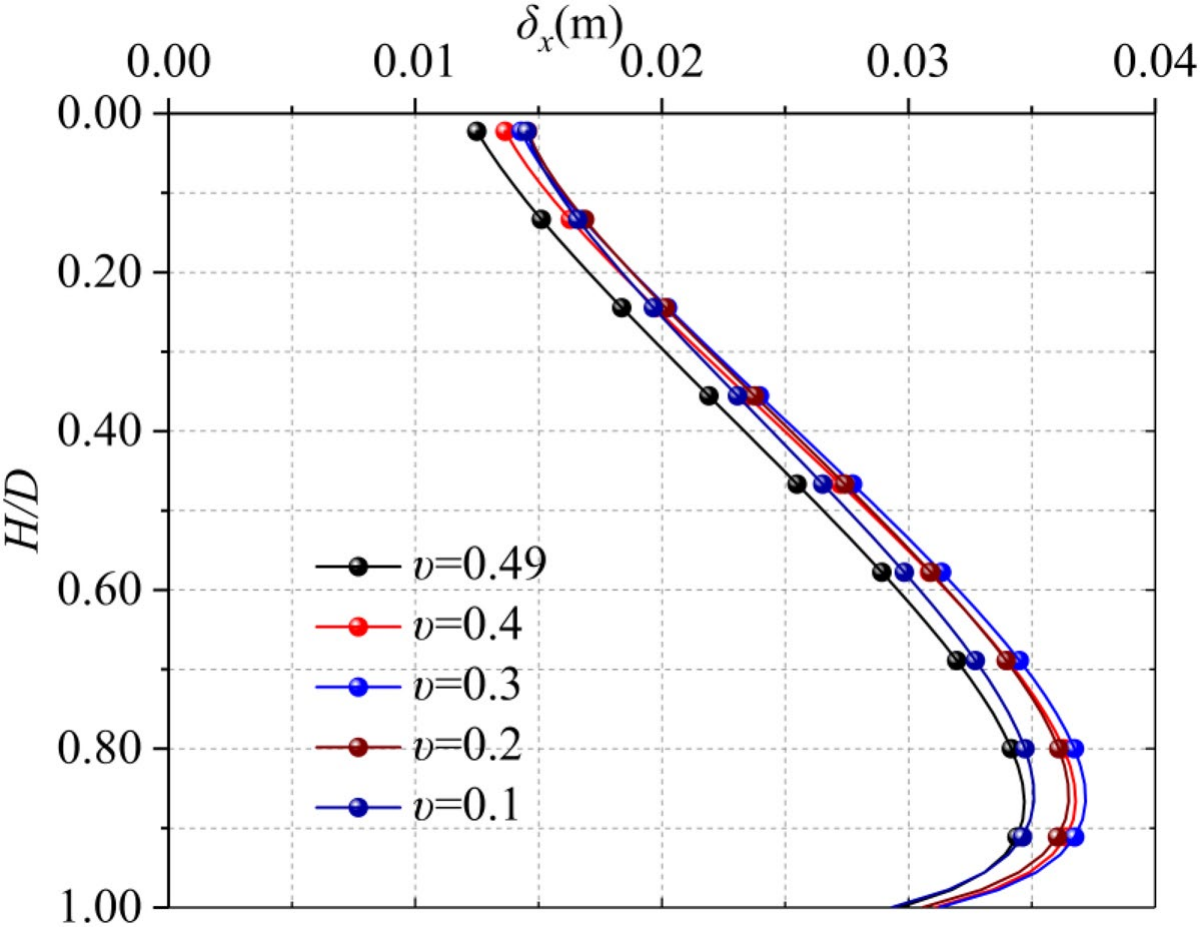
Horizontal displacement of different Poisson's ratio soils.

Poisson's ratio of soil is the ratio of transverse strain to longitudinal strain. It can be seen from Fig. 7, the maximum horizontal displacement occurs at about 5/6 *D*, the range of maximum horizontal deformation is 34.7–37.7 mm. According to the calculation results in Fig. 7, in a certain range (0.1 < *υ* < 0.5), with the increase of Poisson's ratio, the horizontal displacement of the soil caused by the excavation of diaphragm wall gradually increases, but the difference is not obvious and it does not show strict linear law.In General, the Poisson's ratio is not very sensitive to the horizontal displacement of the soil on the side of the trench.

### Length and depth of the trench

**Figure 8:**
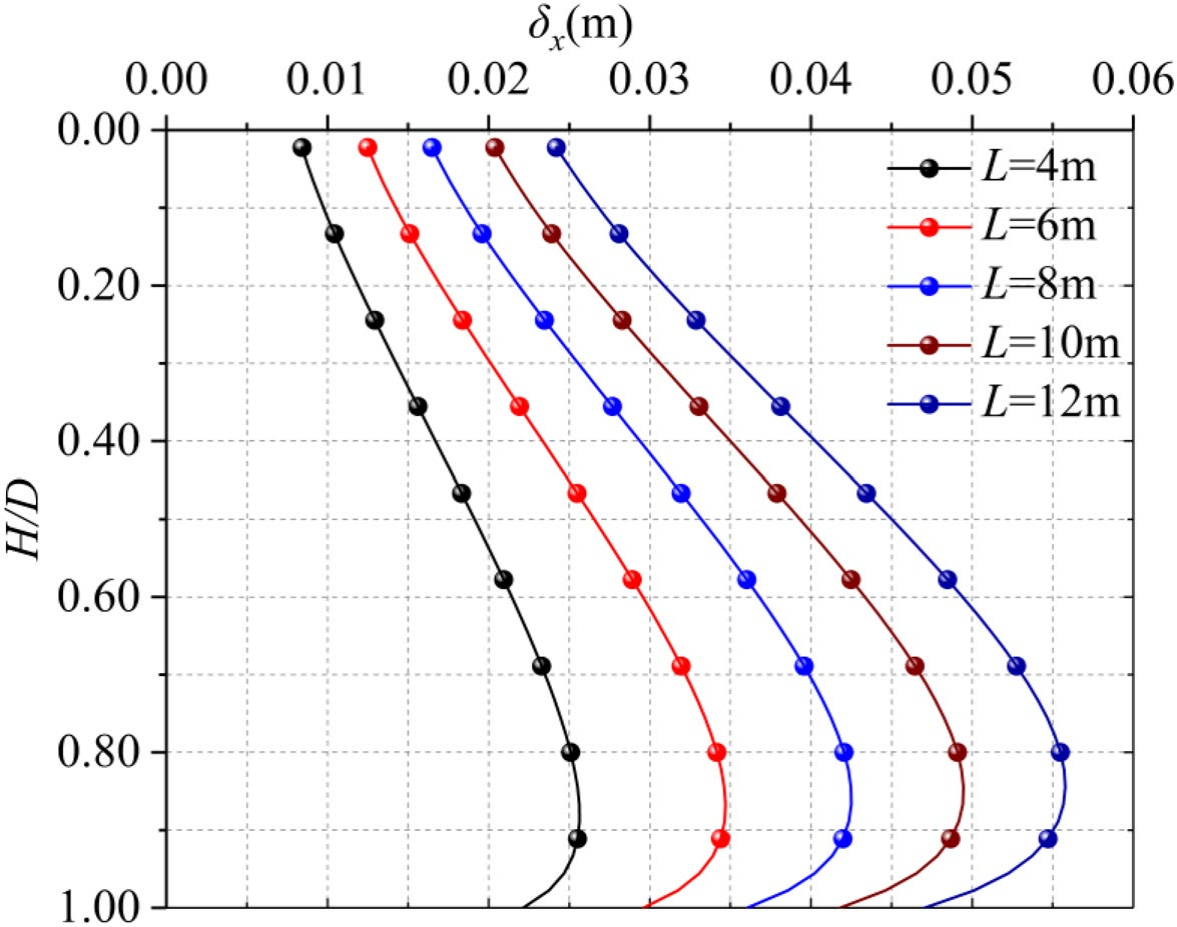
Horizontal displacement of different trench lengths.

**Figure 9:**
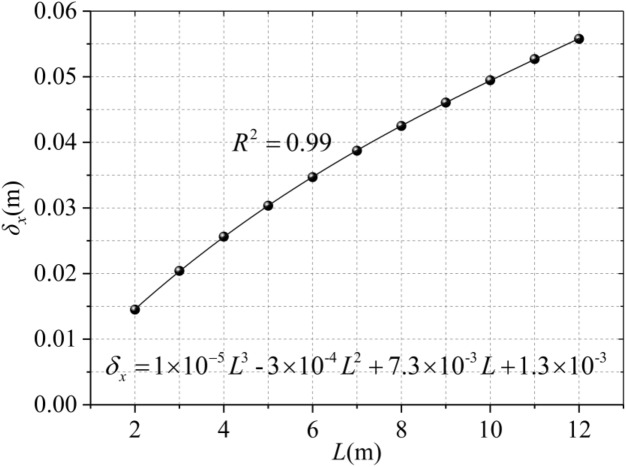
Maximum horizontal displacement of different trench lengths.

The length of a single diaphragm trench has a great influence on the horizontal displacement of the surrounding soil, From the perspective of engineering construction, we hope to increase the length of single diaphragm wall as much as possible, because it can reduce the joint of diaphragm wall and maintain the integrity of diaphragm wall. However, from Fig. 8, it can be seen that for different single diaphragm wall lengths (4 m, 6 m, 8 m, 10 m, 12 m), the longer the single diaphragm wall length is, the greater the horizontal displacement of the soil will be. At the same time, it can be seen that the position where the maximum horizontal displacement occurs is the same (5/6*D*). The increasing rates of the maximum horizontal displacement of the soil caused by each length of a single diaphragm wall are 26.2%, 18.35%, 14% and 11.23%, which conforms to the three-term curve distribution as shown in Fig. 9 and shown in Eq. (13).

13 $$ \delta_{x} = \, 1 \times 10^{ - 5} L^{3} \, - \, 3 \times 10^{ - 4} L^{2} \, + \, 7.3 \times 10^{ - 3} L \, + \, 1.3 \times 10^{ - 3} $$

It can be seen that when the length of a single diaphragm wall is short, with the increase of length, the increase of horizontal displacement of soil is larger (the maximum amplitude can reach 40%), but when the length exceeds a certain length, the increase is smaller. Therefore, we should not only consider the integrity of the diaphragm wall, but also consider the aggravation of soil disturbance caused by the excavation of too long trench. According to the specific situation of the project, we should choose the appropriate trench length.

**Figure 10:**
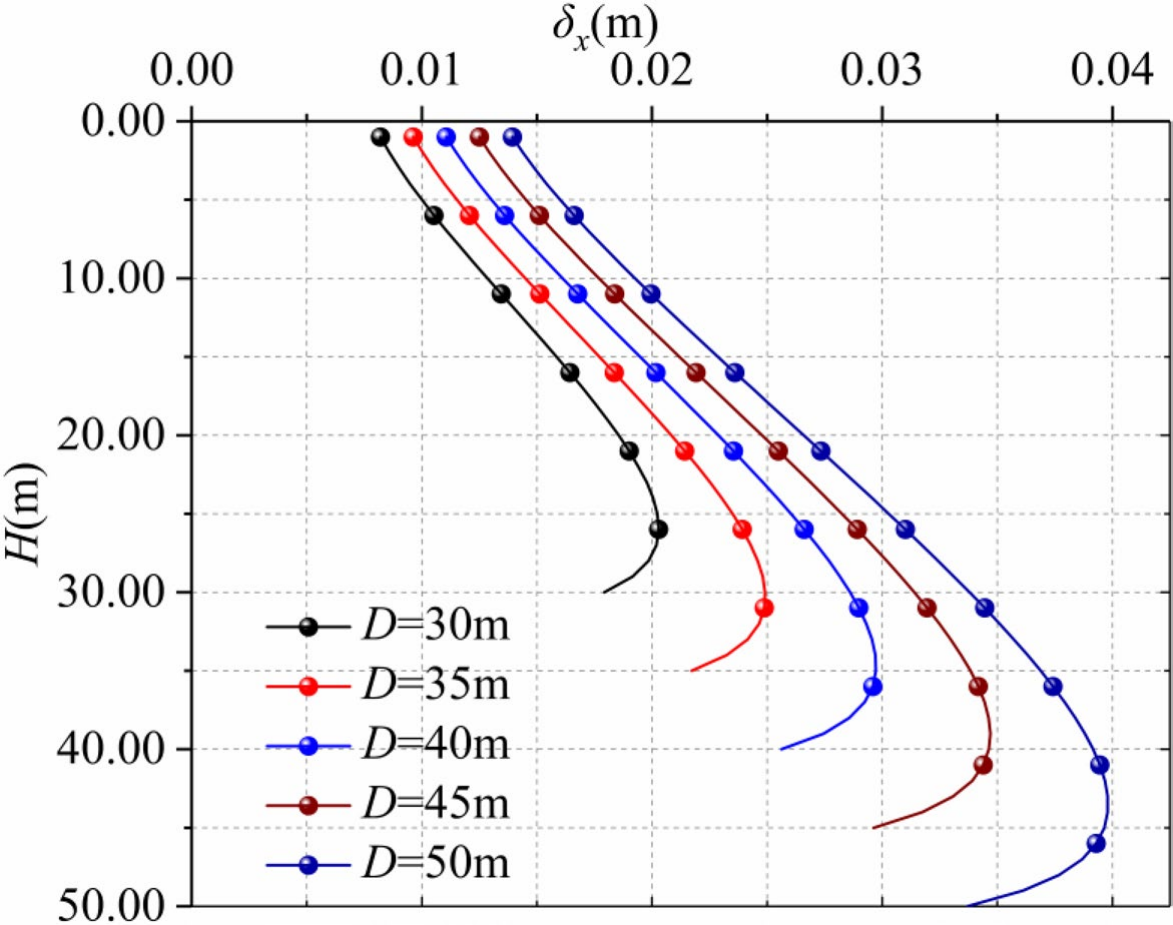
Horizontal displacement of different excavation depths.

**Figure 11:**
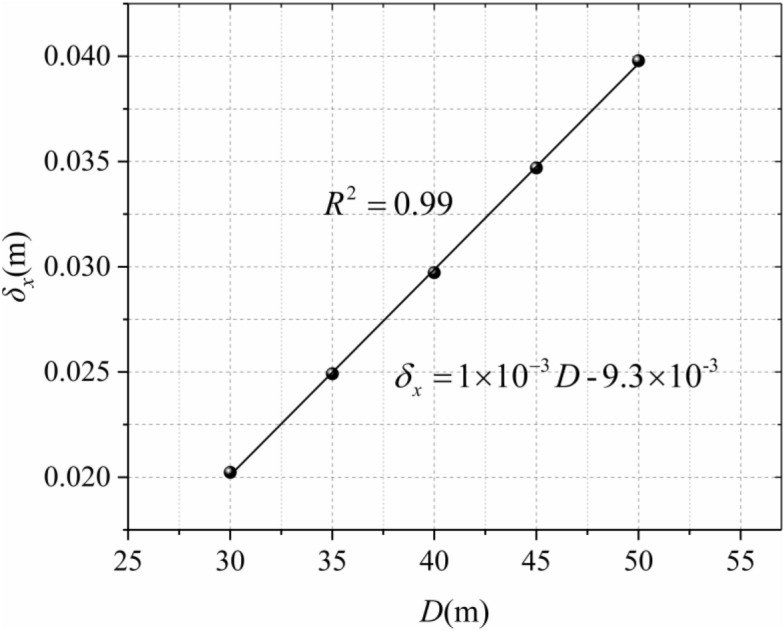
Maximum horizontal displacement of different excavation depths.

The depth of the trench also determines the extent of disturbance to the soil, it can be seen from Fig. 10 that the deeper the trench is, the greater the disturbance to the soil, and the greater the horizontal displacement of the soil around the trench wall. The location of the maximum horizontal displacement is similar (5/6*D*), because Mindlin analytical solution is based on semi-infinite elastic stratum, so the maximum horizontal displacement of each excavation depth is also linear with the groove depth (Fig. 11). It can be observed in the variation range that the depth of the trench is more sensitive to the horizontal displacement of the soil.

### distance from the trench and center line

**Figure 12:**
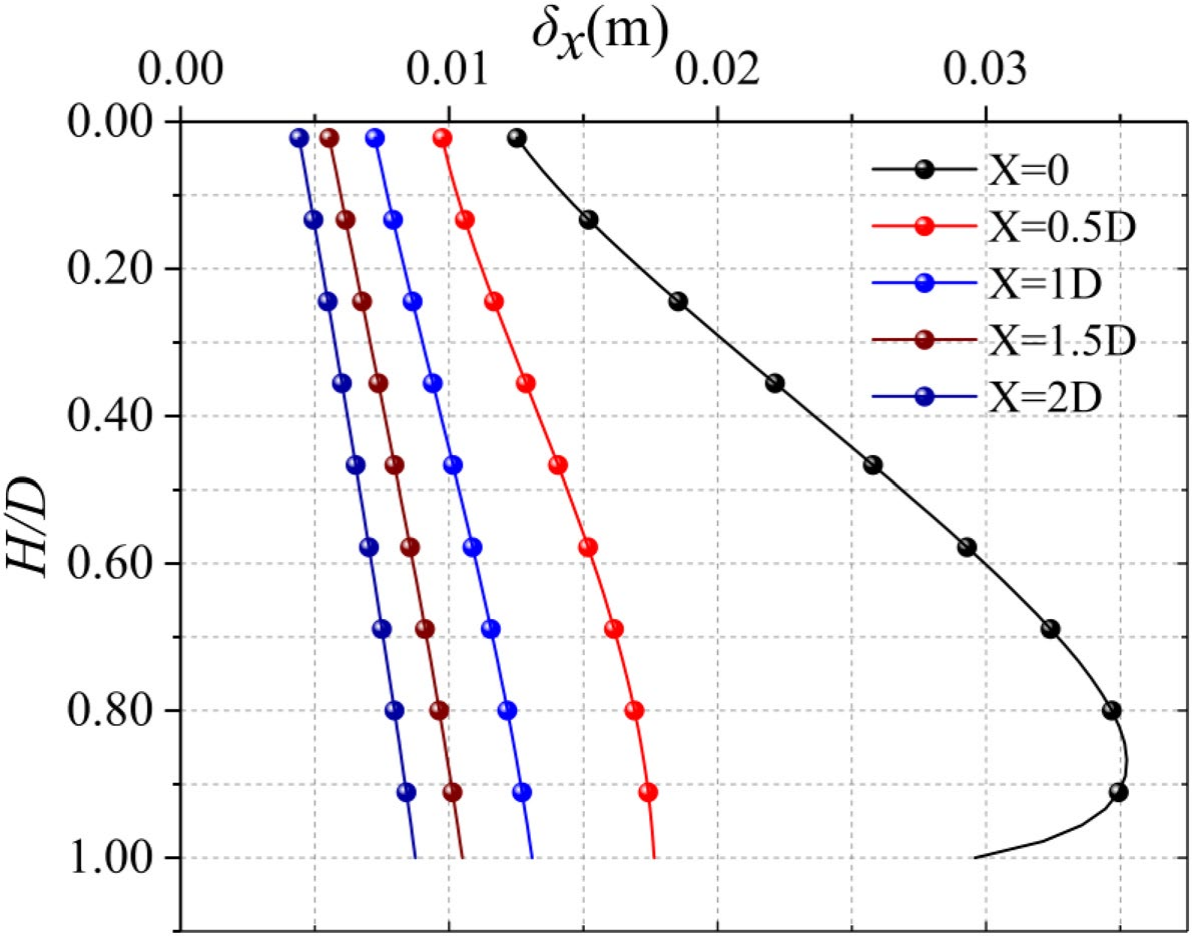
Horizontal displacement at different distances from the trench.

It can be seen from Fig. 12 that the horizontal displacement of the soil closer to the trench is larger. However, the deviation of the maximum horizontal displacement of the soil caused by the distance difference is not obvious.

**Figure 13:**
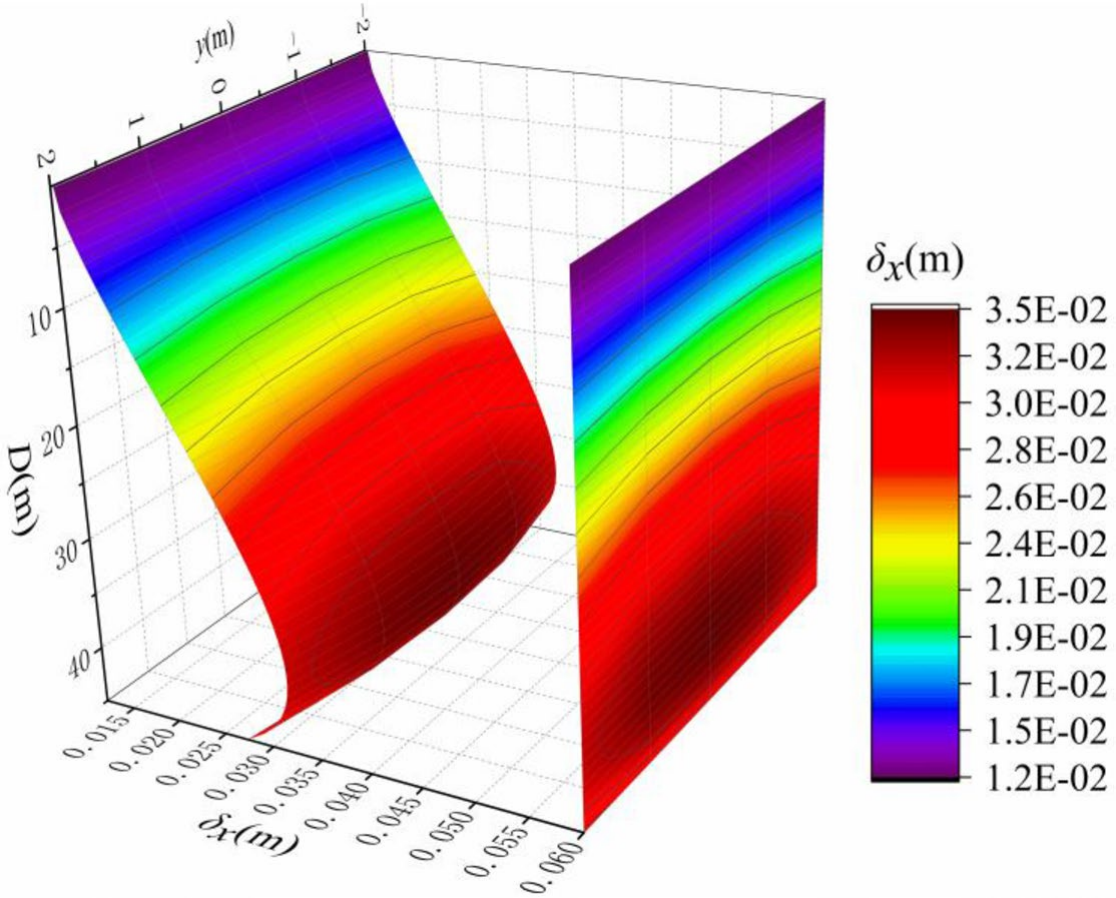
Horizontal displacement at different distances from the centerline of the trench.

**Figure 14:**
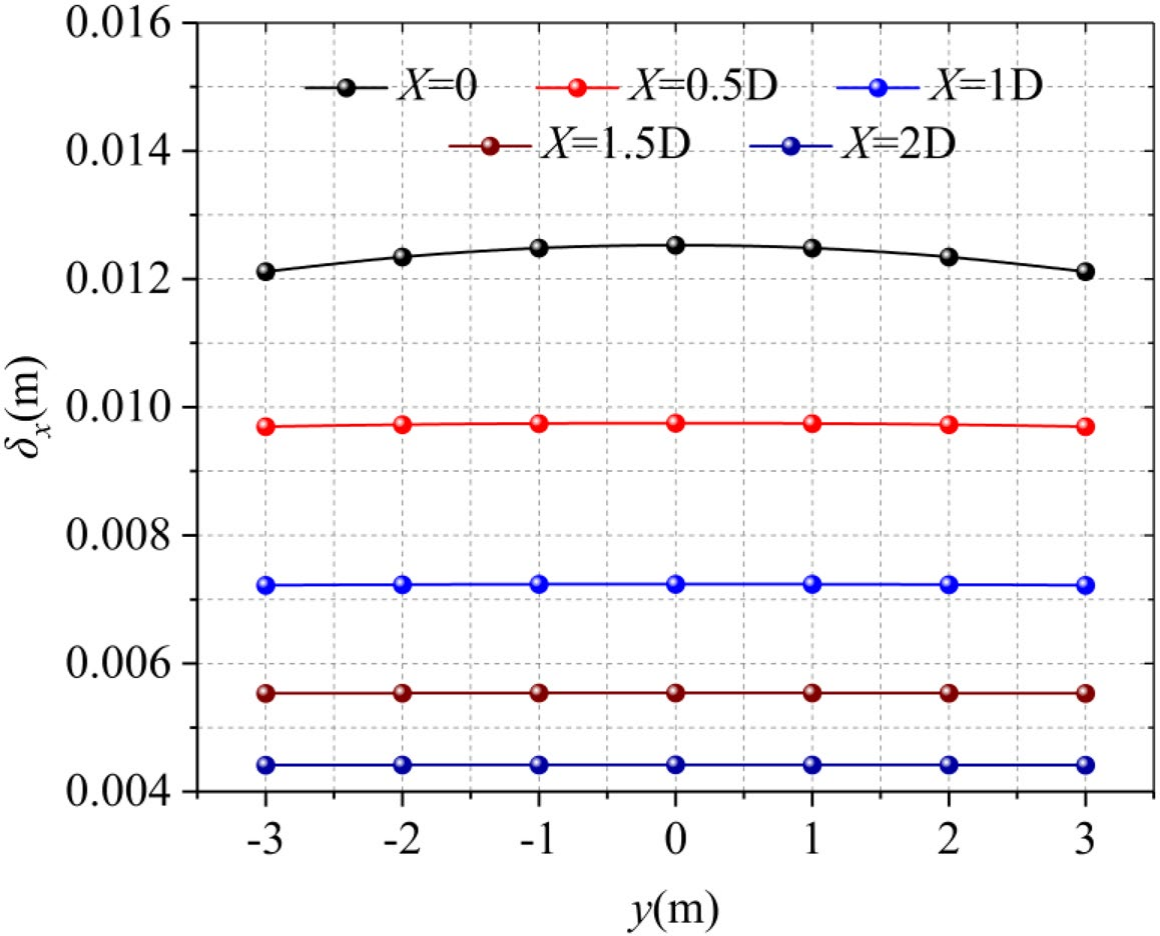
Horizontal displacement at different distances from the trench (H = 0 m).

The horizontal deformation of the soil at the central axis of the excavation trench is shown in Fig. 13, the horizontal displacement of the soil here is the largest, gradually decreasing to both sides, as shown in Fig. 14.

**Figure 15:**
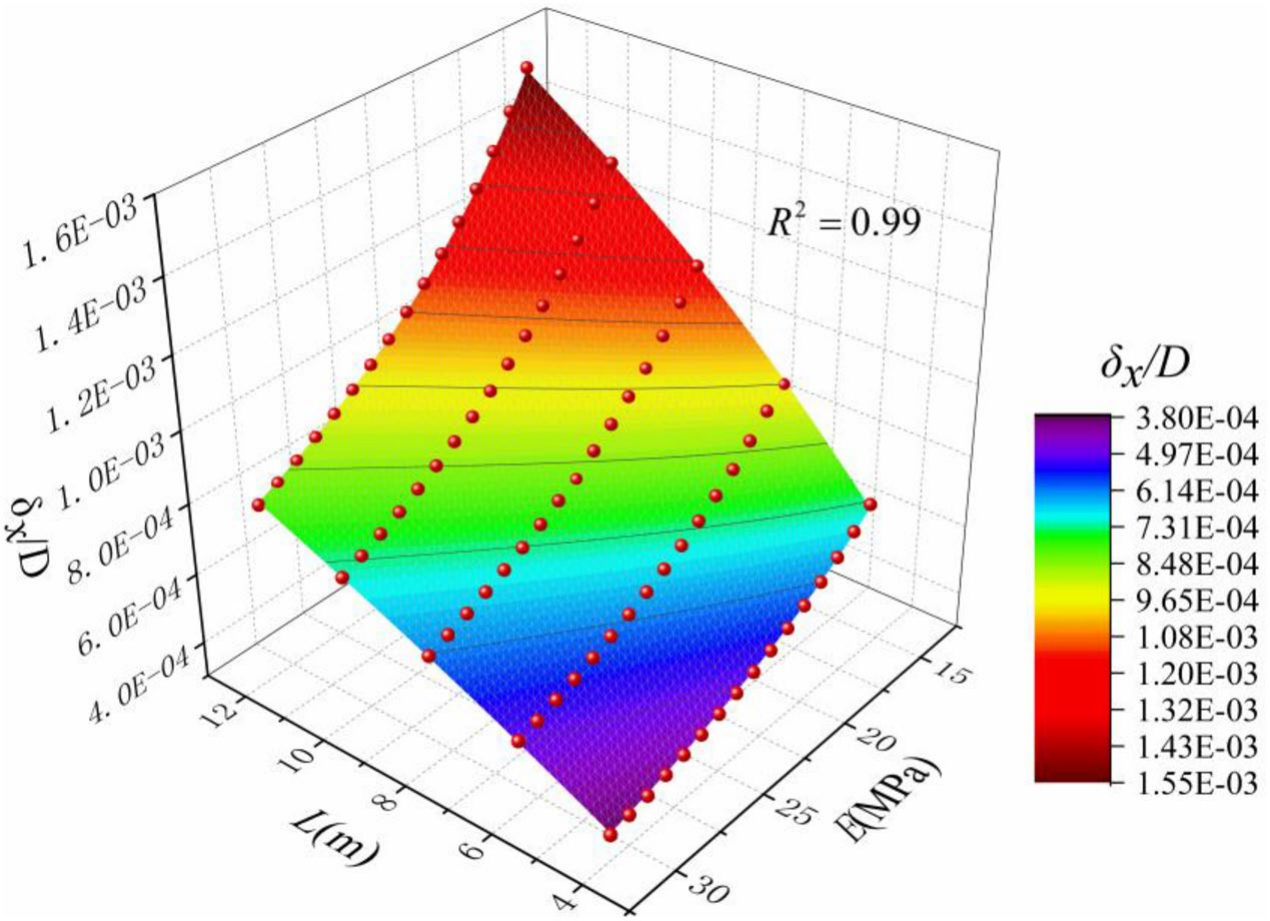
Maximum horizontal displacement based on impact of *E*, *D*, and *L.*

It can be seen from the above that the sensitivity of different distance from the trench and Poisson's ratio to the horizontal displacement of soil is weak, and the horizontal displacement of soil at the central axis of the excavation trench section is the largest, so this position is the most typical, The sensitivity of excavation depth *D*, elastic modulus *E* and length of the trench *L* are strong, so they have great influence on the horizontal displacement of the soil. The maximum horizontal displacement of the soil is linearly related to the excavation depth of the trench, so make it dimensionless (*δ*_*x*_*/D*). Coupling the influence of *D*, *E* and *L* on the maximum horizontal displacement of the soil, the comprehensive influence on the horizontal displacement of the soil is obtained as shown in Fig. 15, and the calculation equation about *E*, *L* and (*δ*_*x*_*/D*) is fitted, as shown in Eq. (14):

14 $$ {{\delta_{x} } \mathord{\left/ {\vphantom {{\delta_{x} } D}} \right. \kern-\nulldelimiterspace} D} = \frac{{m_{1} E^{2} + m_{2} E^{3} }}{{m_{3} L^{2} + m_{4} L^{3} + m_{5} E^{2} }} $$

The coefficients in the equation are shown in Table 1:

**Table 1:** Eq. (14) coefficient.

Parameter	Value	Parameter	Value
m_1_	− 7.86E2	m_2_	8.88
m_3_	4.70E6	m_4_	− 1.44E5
m_5_	− 9.91E4	–	–

Equation (14) is simple in calculation and can be used to calculate the horizontal displacement of soil caused by diaphragm excavation in related cases.

## Sensitivity factors of vertical displacement

The research on the sensitivity factors of vertical displacement of soil around the trench has been mature^13,23–28^. This paper also obtains similar conclusions based on Mindlin analytical solution, which proves the correctness of the analytical solution, and simply summarizes the sensitivity factors of vertical displacement as follows.

**Figure 16:**
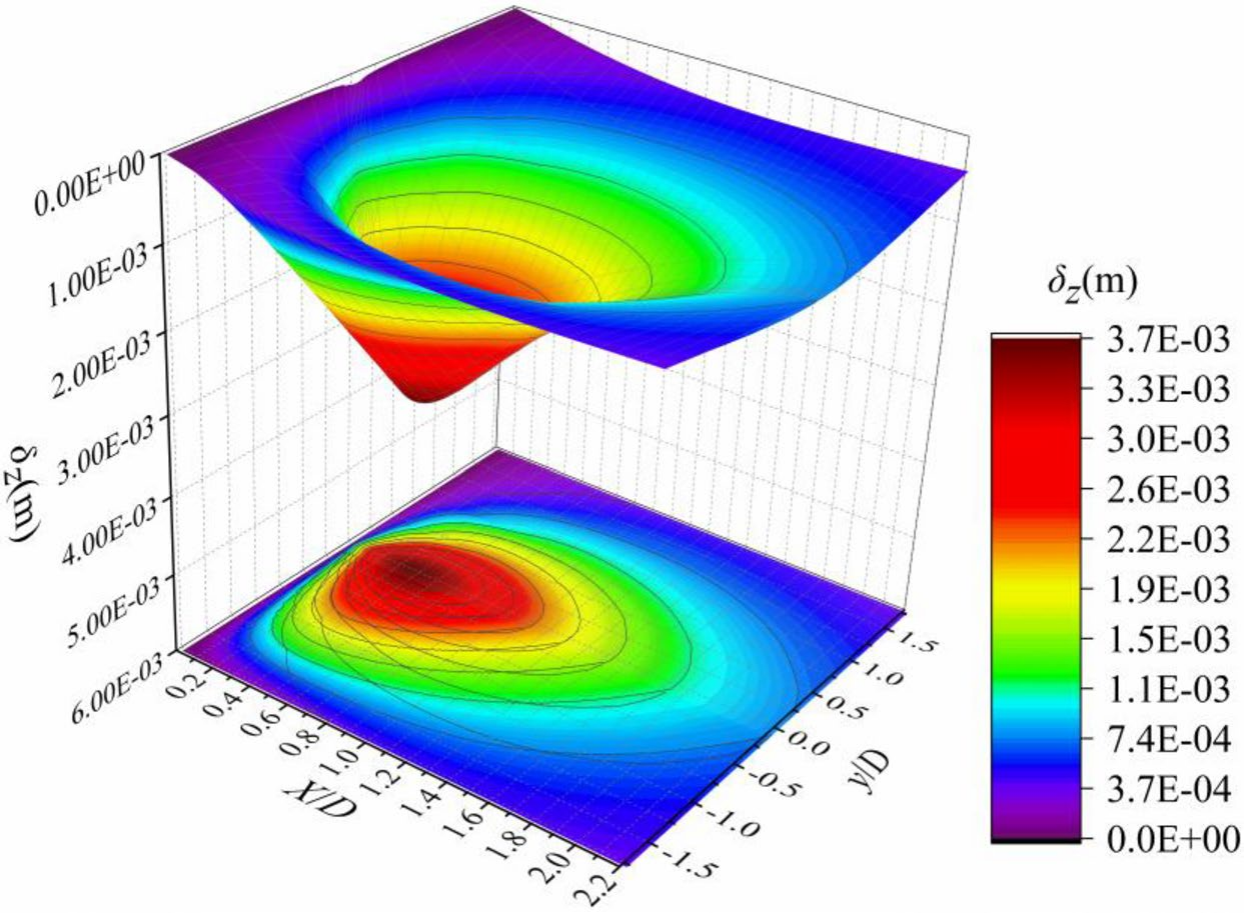
Three-dimensional diagram of the settlement of the surface soil.

It can be seen from Fig. 16 that the vertical displacement of the ground surface around the diaphragm wall trench is like a funnel, the maximum value is located in the excavation center line of the trench, the settlement curve is spoon shaped, and the maximum settlement occurs at a certain distance from the trench.

## Influence of excavation process

The displacement of soil around the trench is also affected by the construction process of concrete diaphragm wall, such as step-by-step excavation, multiple wall construction, etc.

### Step by step excavation

Mindlin solution does not consider the case of stepwise excavation when solving the deformation caused by excavation of the soil, so no matter which layer of excavation has an impact on the soil within the whole excavation depth range, which is different from the actual situation, because when excavating the upper soil, the lower soil will not deform due to the restriction of surrounding soil because it has not been excavated yet.

**Figure 17:**
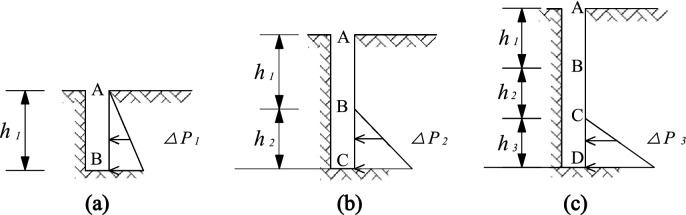
Diagram of step-by-step excavation.

Taking the horizontal displacement of the soil around the trench as an example, considering the influence of step-by-step excavation, the excavation condition of diaphragm wall is simulated from the mechanical point of view, assuming that the whole excavation process is carried out in three steps, namely AB, BC and CD sections, as shown in Fig. 17.

After the AB section excavated, the soil is affected by the hydrostatic pressure of the slurry and the lateral static earth pressure. Because the hydrostatic pressure of slurry is less than the lateral static earth pressure, the soil will deform laterally towards the free surface. At this time, BC and CD sections have not been excavated and will not deform. Therefore, the horizontal displacement of the three sections is respectively:

AB: $$\delta_{x} (AB) = \psi_{11} (z)$$, *0* < *z* < *h*_*1*_;

BC: $$\delta_{x} (BC) = 0$$, *h*_*1*_ < *z* < *h*_*1*_ + *h*_*2*_;

CD: $$\delta_{x} (CD) = 0$$, *h*_*1*_ + *h*_*2*_ < *z* < *h*_*1*_ + *h*_*2*_ + *h*_*3*_。

$\delta_{x} (CD)$: horizontal displacement of CD section, $\psi_{ij} (z)$: horizontal displacement of *j* section in the depth range *z* caused by excavation of step *i*.

The second step is to excavate the BC section. At this time, the deformation of AB section tends to be stable due to the stress release. The excavation of BC section increased the unbalanced force, resulting in the secondary deformation of the soil within the depth of 0 to *h*_*2*_. The horizontal displacements of the three sections are as follows:

AB: $$\delta_{x} (AB) = \psi_{11} (z) + \psi_{21} (z)$$, *0* < *z* < *h*_*1*_;

BC: $$\delta_{x} (BC) = \psi_{22} (z)$$, *h*_*1*_ < *z* < *h*_*1*_ + *h*_*2*_;

CD: $$\delta_{x} (CD) = 0$$, *h*_*1*_ + *h*_*2*_ < *z* < *h*_*1*_ + *h*_*2*_ + *h*_*3*_。

Finally, the third step of excavation is completed, which causes the third disturbance to the soil. At this time, the horizontal displacement of the three sections is respectively:

AB:$$\delta_{x} (AB) = \psi_{11} (z) + \psi_{21} (z) + \psi_{31} (z)$$, *0* < *z* < *h*_*1*_;

BC:$$\delta_{x} (BC) = \psi_{22} (z) + \psi_{32} (z)$$, *h*_*1*_ < *z* < *h*_*1*_ + *h*_*2*_;

CD:$$\delta_{x} (CD) = \psi_{33} (z)$$, *h*_*1*_ + *h*_*2*_ < *z* < *h*_*1*_ + *h*_*2*_ + *h*_*3*_。

**Figure 18:**
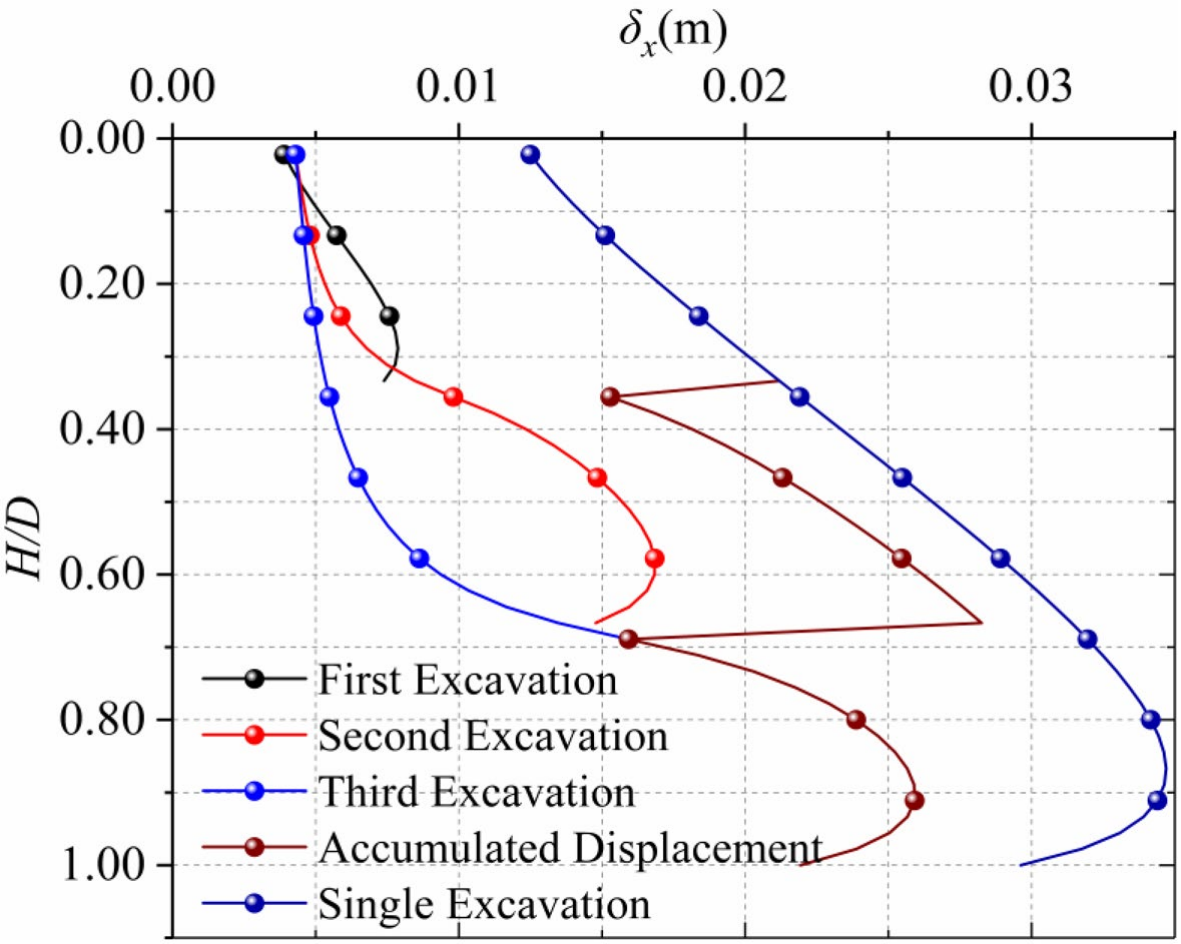
Comparison of horizontal displacement of step-by-step excavation and one-time excavation.

**Figure 19:**
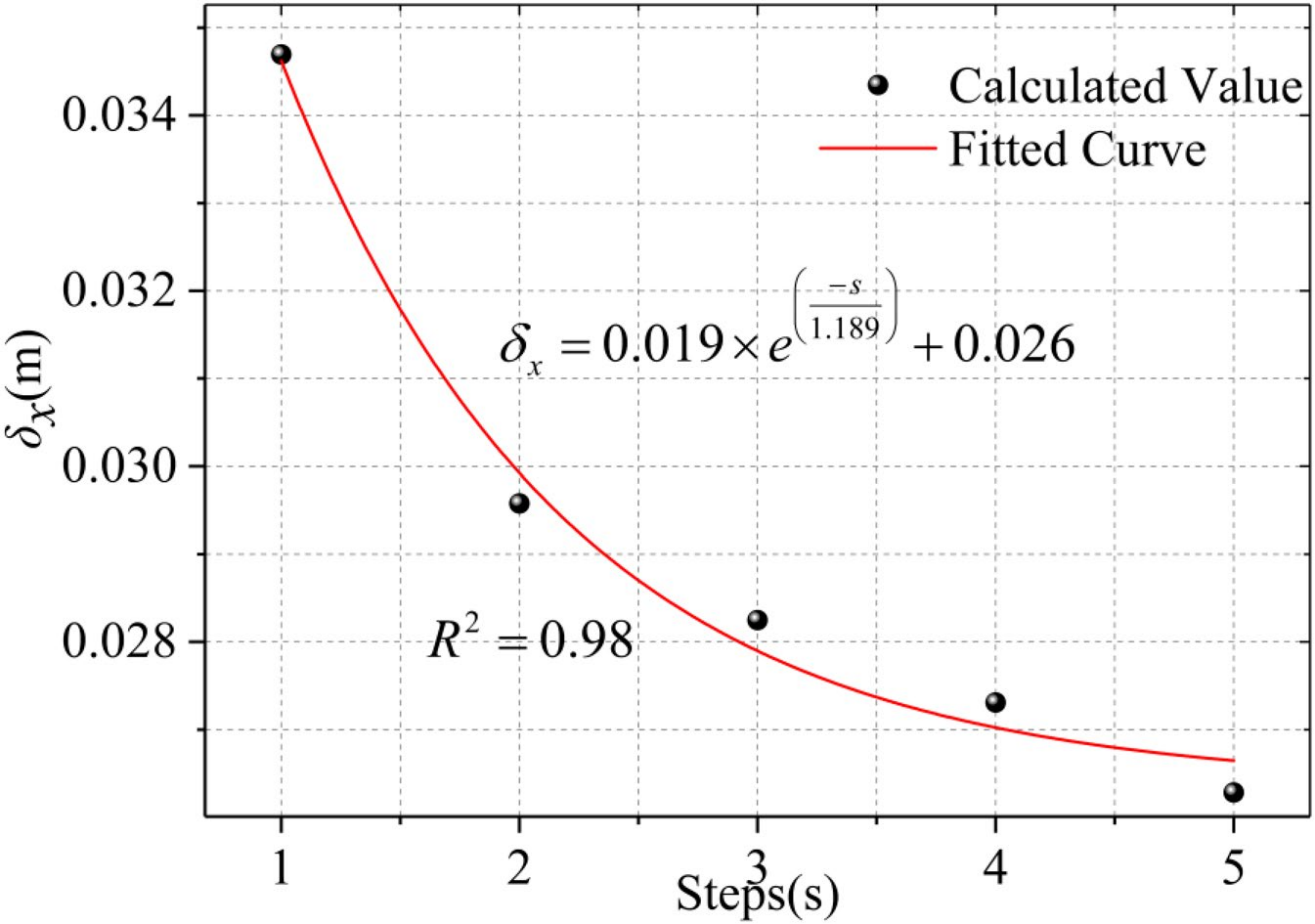
Relationship between step-by-step excavation steps and maximum horizontal displacement.

Calculated with the parameters selected in Sect. 2.3, the results are shown in Fig. 18, The accumulated horizontal displacement of soil caused by step excavation is smaller than the displacement caused by one-time excavation calculated by Mindlin analytical solution. It can be seen from Fig. 19 that with the increase of excavation steps, the horizontal displacement of the soil around the trench decreases and tends to be stable. The relationship between excavation steps and the maximum horizontal displacement is quadratic polynomial. Excavation of diaphragm wall is a dynamic process, in fact, it is split into countless steps. Depending on the law of the curve, we can roughly infer that the maximum horizontal displacement in the actual process of diaphragm wall excavation can be reduced by about 30% based on the horizontal displacement of one excavation trench obtained by Mindlin analytical solution.

### influence of multiple walls

**Figure 20:**
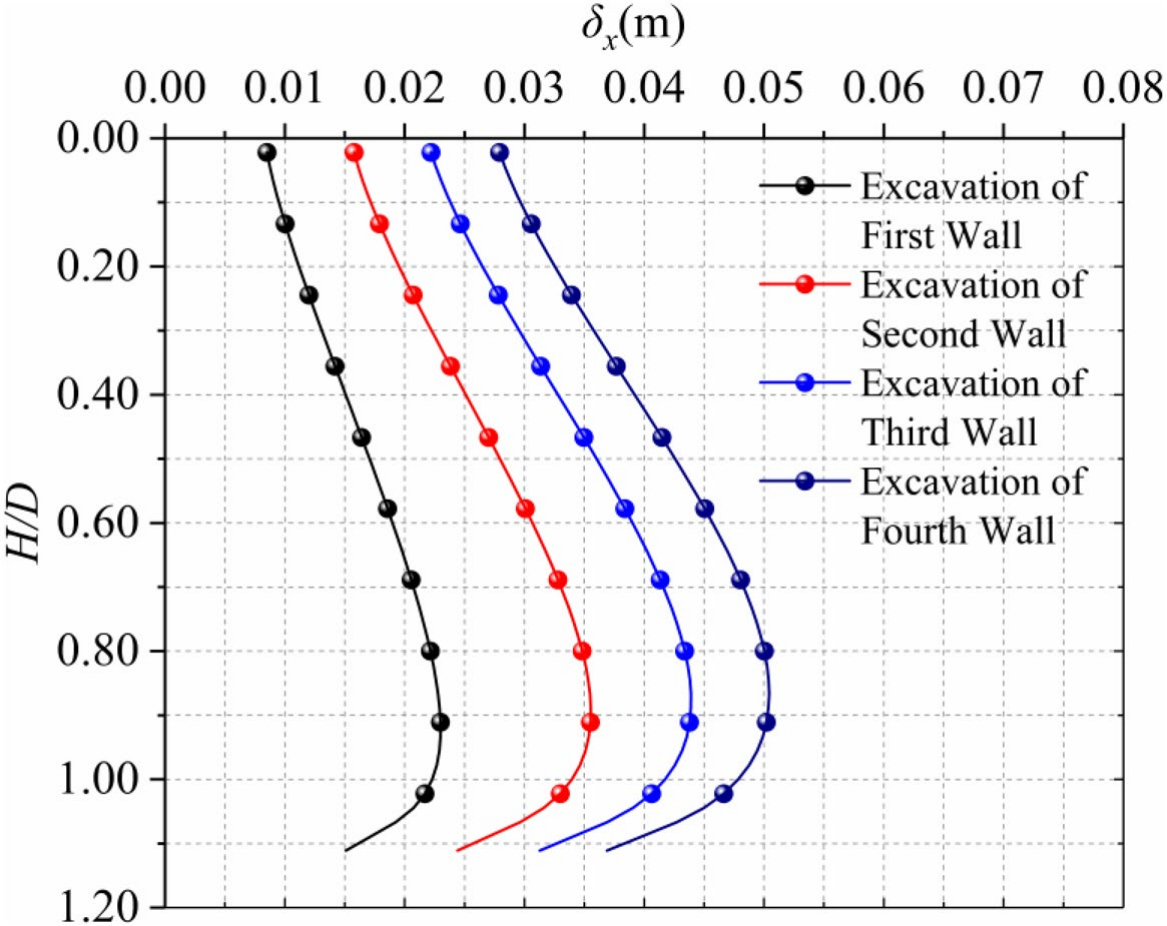
Horizontal displacement caused by multiple trench excavation.

The diaphragm wall is connected by each wall through joints, so it is necessary to consider the construction impact of multiple walls, taking the central axis of the first trench section as an example, it can be seen from Fig. 20 that the horizontal displacement of the soil during the construction of this wall is the largest, for example, without considering the influence of the concrete pouring of the diaphragm wall, When excavating to the adjacent second wall, the disturbance of the soil has been greatly reduced, and the horizontal displacement generated at this time is about 55% of it of the first wall. When excavating to the adjacent third wall and the fourth wall, the horizontal displacement is 37% and 29% of that. This solution does not take into consideration the reinforcement and displacement limitation of the existing diaphragm wall to the stratum. It can be seen that although there is a certain horizontal displacement, it has little effect on the overall stability.

## Conclusion

Based on Mindlin solution, an analytical solution is proposed to calculate the soil deformation caused by the formation of the diaphragm wall under the slurry retaining wall. The calculated results agree well with the measured values and have a strong applicability. From the above analysis results, we can see that:

1. The horizontal displacement of the surrounding soil caused by the trench excavation of diaphragm wall increases with the increase of the depth, and the extreme value appears at the depth of about 5/6*D*, then decreases sharply; the horizontal displacement is spoon shaped, and the distance between the maximum settlement point and the trench increases with the increase of the *D* and *υ.*
2. Among the calculation parameters, the three parameters of excavation *D*, *E* and *L* have strong sensitivity and great influence on the soil displacement, and a simple calculation equation of horizontal displacement based on *E*, *D* and *L* is derived.

However, the calculation results in this paper are different from those in some projects, which may be because the soil is a homogeneous semi-infinite space body by default in the process of solution in this paper, and its elastic modulus is exactly the same along the depth direction, which is not consistent with the real stratum situation. The actual stratum usually has different soil layers with different mechanical properties, even if it is the same soil layer, its elastic modulus also changes with the depth Change.
